# Recombinant phospholipase A1 (Ves v 1) from yellow jacket venom for improved diagnosis of hymenoptera venom hypersensitivity

**DOI:** 10.1186/1476-7961-8-7

**Published:** 2010-04-01

**Authors:** Henning Seismann, Simon Blank, Liliana Cifuentes, Ingke Braren, Reinhard Bredehorst, Thomas Grunwald, Markus Ollert, Edzard Spillner

**Affiliations:** 1 Institute of Biochemistry and Molecular Biology, Department of Chemistry, University of Hamburg, Germany; 2 PLS-Design GmbH, Hamburg, Germany; 3 Clinical Research Division of Molecular and Clinical Allergotoxicology, Department of Dermatology and Allergy, Technische Universität München, Germany

## Abstract

**Background:**

Hymenoptera venoms are known to cause life-threatening IgE-mediated anaphylactic reactions in allergic individuals. Proper diagnosis of hymenoptera venom allergy using venom extracts is severely affected by molecular cross-reactivities. Although non-glycosylated marker allergens would facilitate the identification of the culprit venom, the major allergen phospholipase A1 (Ves v 1) from yellow jacket venom (YJV) remained unavailable so far.

**Methods:**

Expression of Ves v 1 as wild type and enzymatically inactivated mutant and Ves v 5 in insect cells yielded soluble proteins that were purified via affinity chromatography. Functionality of the recombinant allergens was assessed by enzymatic and biophysical analyses as well as basophil activation tests. Diagnostic relevance was addressed by ELISA-based analyses of sera of YJV-sensitized patients.

**Results:**

Both major allergens Ves v 1 and Ves v 5 could be produced in insect cells in secreted soluble form. The recombinant proteins exhibited their particular biochemical and functional characteristics and were capable for activation of human basophils. Assessment of IgE reactivity of sera of YJV-sensitized and double-sensitized patients emphasised the relevance of Ves v 1 in hymenoptera venom allergy. In contrast to the use of singular molecules the combined use of both molecules enabled a reliable assignment of sensitisation to YJV for more than 90% of double-sensitised patients.

**Conclusions:**

The recombinant availability of Ves v 1 from yellow jacket venom will contribute to a more detailed understanding of the molecular and allergological mechanisms of insect venoms and may provide a valuable tool for diagnostic and therapeutic approaches in hymenoptera venom allergy.

## Background

Hymenoptera stings may cause life-threatening and sometimes fatal IgE-mediated anaphylactic reactions with the major threat emanating from the yellow jacket *V. vulgaris *and the honeybee *A. mellifera*. Although venom immunotherapy is highly effective, an adequate diagnosis and identification of the culprit venom is hampered by the use of crude venoms for measurement of specific IgE levels. Thereby, the main problem arises from serologic double-positivity for *A. mellifera *and *V. vulgaris *venom of up to 50% of patients that have IgE against hymenoptera venoms [1]. Apart from true double-sensitisation this phenomenon is largely attributed to molecular cross-reactivity, either based on the presence of cross-reactive epitopes in homologues proteins of both venoms such as the hyaluronidases and dipeptidylpeptidases, or the presence of so-called cross-reactive carbohydrate determinants (CCD) which account for 70-80% of cross-reactive patients found within the double-positive cohort [2].

Since common diagnostic approaches are not capable of circumventing or differentiating these cross-reactivities considerable interest has emerged in strategies enabling an improved diagnosis. Fail-safe identification of the culprit venom is imperative for design of appropriate therapeutic intervention with either one or both venoms and thus key to any improvement.

The most promising approach for the development of reliable diagnostics as well as safer and more efficacious patient-tailored treatment modalities relies on the use of defined recombinant allergens [3]. For honeybee venom (HBV), phospholipase A2 (Api m 1) has emerged as surrogate marker, however, for YJV access to native proteins is limited and only a minor number of recombinant allergens are available [4,5].

The three yellow jacket allergens thought primarily responsible for IgE-mediated allergic reactions include phospholipase A_1 _(Ves v 1), hyaluronidase (Ves v 2), and antigen 5 (Ves v 5) [6]. To some extent this estimation is prompted by the concentration of the particular proteins in the venom. However, the relevance of Ves v 2 as allergen has been disregarded [7], while the novel high molecular weight compound Ves v 3 recently was reported to exhibit a pronounced allergenic potential [8]. Both Ves v 2 and Ves v 3 are glycoproteins prone to CCD reactivity with homologues in HBV.

By contrast, Ves v 1 and Ves v 5 are non-glycosylated and unique candidates for diagnosis of YJV allergy. While expression of Ves v 5 could be demonstrated in various eu- and prokaryotic hosts [9], only insoluble protein was obtained in scarce attempts of Ves v 1 expression [10] rendering a reliable assessment of IgE reactivities on the basis of such a protein questionable.

In this study, we report the successful expression of both, Ves v 1 and Ves v 5, in insect cells and their subsequent biochemical and immunological characterisation. The unanticipatedly pronounced prevalence of IgE reactivity in YJV-sensitised patients to rVes v 1 emphasises the need for two unique recombinant major allergens from YJV, especially in terms of double-positivity.

## Methods

### Materials

Three groups of sera were selected at random from the institutional serum bank: (i) Sera with a positive sIgE test to HBV (i1 ≥0.1 kUa/L) and YJV (i3 ≥ 0.1 kUa/L) (n = 20); (ii) Sera with a positive sIgE test to YJV only (i3 ≥ 0.1 kUa/L) (n = 14); (iii) Sera with a positive sIgE test to HBV only (i1 ≥ 0.1 kUa/L) (n = 5). All patients had given their informed written consent to draw an additional serum sample.

### Cloning of cDNA

Total RNA was isolated from yellow jacket (*Vespula vulgaris*) venom sacks using peqGold TriFast™ (Peqlab Biotechnologie, Erlangen, Germany). SuperScript III Reverse Transcriptase (Invitrogen, Karlsruhe, Germany) was used to synthesize cDNA. Full length Ves v 1 was amplified with *Pfu *DNA polymerase (Fermentas, St. Leon-Rot, Germany) using the primers 5'-GGACCCAAATGTCCTTTTAATTC-3' and 5'-AACCGCGGTTAAATTATCTTCCCCTTGTTA-3'. Full length Ves v 5 was amplified employing the primers 5'-AACAATTATTGTAAAATAAAATGTTTGAAA-3' and 5'-CTTTGTTTGATAAAGTTCCT-3'. An N-terminal 10-fold His-tag and a V5 epitope as well as 5' BamHI and 3' NotI restriction sites were added by PCR and the PCR product was subcloned into the pAcGP67-B baculovirus transfer vector (BD Pharmingen, Heidelberg, Germany) after restriction digest with BamHI and NotI.

### Site directed mutagenesis

For generation of an inactive Ves v 1 form two amino acid residues in the potential active site were altered by using the QuikChange Site directed mutagenesis Kit (Stratagen, La Jolla, USA) according to the manufacturers' recommendations of the employing the primers 5'-CGATTAATTGGACATGGCTTAGGAGCACATG-3' and 5'-CATGTGCTCCTAAGCCATGTCCAATTAATCG-3' for S137G exchange and 5'-GAAATTATTGGGCTTGCTCCTGCTAGGCCTT-3' and 5'-AAGGCCTAGCAGGAGCAAGCCCAATAATTTC-3' for N165A exchange.

### Recombinant baculovirus production and expression

Recombinant baculovirus was generated by cotransfection of *Spodoptera frugiperda *(Sf9) cells (Invitrogen) with BaculoGold bright DNA (BD Pharmingen) and the baculovirus transfer vector pAcGP67-B Ves v 1 or Ves v 5, respectively, according to recommendations of the manufacturer. High titer stocks were produced by three rounds of virus amplification and optimal multiplicity of infection (MOI) for recombinant protein expression was determined empirically by infection of Sf9 cells with serial dilutions of high titer virus stock.

### Expression in baculovirus-infected Sf9 cells

High titer stocks of recombinant baculovirus containing the Ves v 1 or Ves v 5 coding DNA were used to infect Sf9 cells (1.5-2.0 × 10^6 ^cells per ml) in a 2000 ml suspension flask (400 ml suspension culture). For protein production the cells were incubated at 27°C and 110 rpm for 72 h.

### Protein purification

Cellular supernatants were applied to a nickel-chelating affinity matrix (Ni-NTA-agarose, Qiagen, Hilden, Germany). After washing with NTA-binding buffer (50 mM sodium phosphate, pH 7.6, 500 mM NaCl) the protein was eluted with NTA-binding buffer containing 300 mM imidazole.

### Enzymatic activity of rVes v 1

The enzymatic activity was assessed by use of the EnzChek Phospholipase A1 Assay Kit (Invitrogen) according to the recommendation of the manufacturer.

### Biophysical analysis of rVes v 5

Dynamic light scattering (DLS) of rVes v 5 was performed using a Spectroscatterer 201 (RiNA GmbH, Berlin, Germany) equipped with a He-Ne laser providing radiation with a wavelength of 690 nm and an output power in the range of 10-50 mW. The sample (30 μl) with a protein concentration of 0.12 mg/ml in 50 mM sodium phosphate, 150 mM NaCl, pH 7.6 were placed in a quartz cuvette and measured at a constant temperature of 20°C.

Circular dichroism spectra were recorded at 20°C using a Jasco J-715 spectropolarimeter (Jasco, Groβ-Umstadt, Germany). A 1-mm optical pathlength quartz cell was used to obtain spectra in the far-UV region (190 to 260 nm) at a protein concentration of 0.015 mg/ml in 50 mM sodium phosphate, 150 mM NaCl, pH 7.65 μM. The CD spectra were acquired at a scan speed of 20 nm/min and a step resolution of 0.1 nm.

### Immunoreactivity of human sera

For assessment of specific IgE immunoreactivity of human sera in ELISA, 384 well microtiter plates (Greiner, Frickenhausen, Germany) were coated with recombinant allergen, nApi m 1 (Latoxan, Valence, France) and the CCD marker MUXF-BSA (10 μg/ml) (provided by Siemens Healthcare Diagnostics, Los Angeles, USA) at 4°C overnight and blocked with 40 mg/ml skimmed milk powder in PBS at room temperature. Human sera were diluted 1:2 in PBS and applied for 4 hours at room temperature. Wells were rinsed 4 times with PBS and incubated with a monoclonal alkaline phosphatase-conjugated mouse anti-human IgE antibody (BD Pharmingen, clone G7-26) diluted 1:1000 in 20 mg/ml skimmed milk powder in PBS. Wells were again rinsed 4 times with PBS and substrate solution (5 mg/ml 4-nitrophenylphosphate, AppliChem, Darmstadt, Germany) was added. After 30 minutes plates were read at 405 nm.

### Basophil activation test

The basophil activation test was essentially performed as recommended by the manufacturer (Bühlmann Laboratories, Basel, Switzerland). Stimulation with recombinant allergen was performed at protein concentrations of 0.1, 200 and 2000 ng/ml. YJV at a concentration of 50 ng/ml was used as positive stimulation control served (Bühlmann Laboratories) while plain stimulation buffer was used as negative stimulation control.

### Other methods

SDS-PAGE and Western blotting as well as standard procedures in molecular biology were performed according to established protocols [11].

## Results

### cDNA cloning and recombinant expression in insect cells

For recombinant production of the YJV allergens Ves v 1 and Ves v 5 the particular cDNA was amplified from yellow jacket venom gland cDNA. Ves v 1 was produced as a wild type molecule in order to use the inherent activity as an indicator of proper folding. However, to avoid potentially detrimental effects of this activity on expression yields an additional mutant version of phospholipase A1, Ves v 1 S137G/N165A, lacking phospholipase activity was generated by site directed mutagenesis.

**Figure 1:**
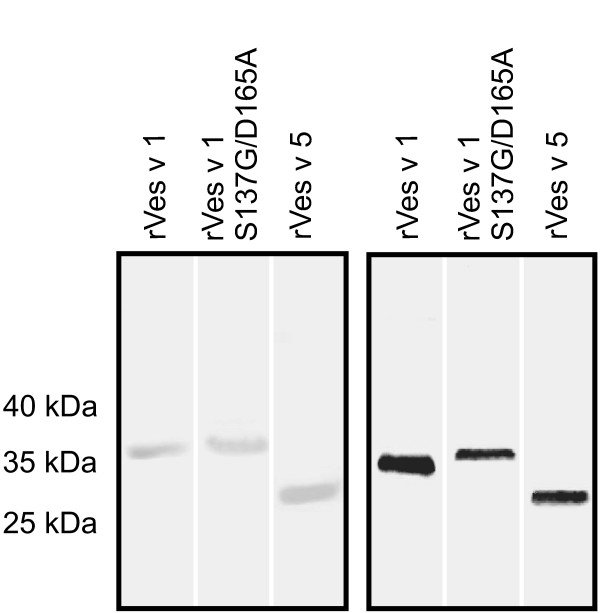
**SDS-PAGE and Immunoblot of rVes v 1 and rVes v 5**. Left panel: SDS-PAGE analysis of recombinant allergens recombinantly produced in Sf9 insect cells visualised by Coomassie Blue staining (lane 1: rVes v 1; lane 2: rVes v 1 S137G/D165A; lane 3: rVes v 5). Right panel: Immunoblot analysis with anti-V5 epitope antibody (lane 1: rVes v 1; lane 2: rVes v 1 S137G/D165A; lane 3: rVes v 5).

Subsequently, all proteins were produced by baculovirus-based infection of eukaryotic Sf9 insect cells and secretion of the proteins into the cellular supernatant. The epitope tagged rVes v 1 and rVes v 5 were obtained with yields of approx. 0.2 μg and 1.5 μg, respectively, per ml of culture supernatant. Thereby, both variants of Ves v 1 could be produced in comparable amounts suggesting that the phospholipase activity exerts no adverse effect on expression. In accordance with the native venom proteins the recombinant analogues exhibited an apparent molecular mass in SDS-PAGE of approx. 37 kDa and 27 kDa (Fig. 1).

### Biochemical characterisation of rVes v 1 and rVes v 5

Prior to an immunological assessment the recombinant allergens were characterised regarding their molecular authenticity. Due to the lack of an inherent enzymatic activity, the physicochemical characteristics of rVes v 5 were analysed by biophysical methods. For *E. coli *derived rVes v 5 tendecies towards oligomerisation have been reported [12]. However, as assessed by DLS measurements (additional file 1) insect cell derived rVes v 5 exhibited clear monodispersity with a hydrodynamic radius of 2.6 +/- 0.4 nm. Furthermore, the structural features of rVes v 5 as assessed by CD spectroscopy (additional file 2) were identical to those reported for nVes v 5 [9].

**Figure 2:**
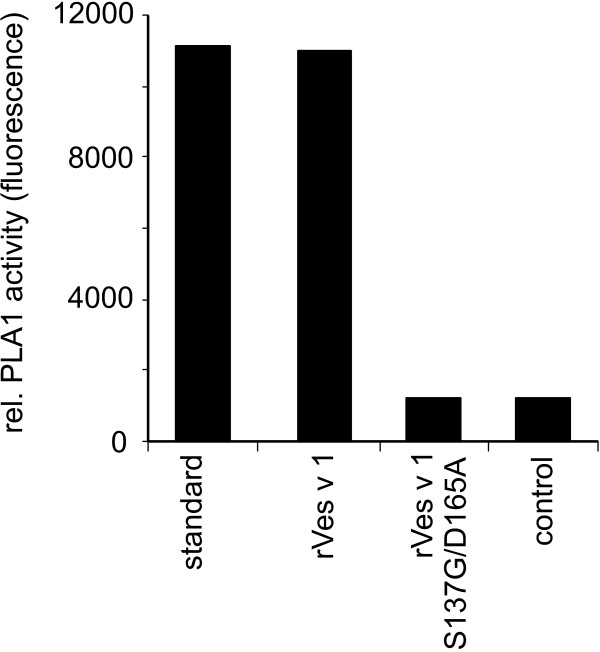
**Enzymatic activity of rVesv 1**. Phospholipase activity assay of rVes v 1 and rVes v 1 S137G/D165A. The recombinant proteins were used at a concentration of 10 μg/ml. Lecithase^® ^ultra at a concentration of 2.5 U/ml was used as a positive control, while the negative control was conducted by omission of protein.

For the rVes v 1 protein, functionality could be addressed by determining the inherent enzymatic activity using a colorimetric phospholipase assay (Fig. 2). The specific activity of the wild type protein was determined to be approx. 2.5 U/ml at a concentration of 10 μg per ml. As anticipated, the mutant rVes v 1 S137G/N165A did not exhibit enzymatic activity. Both, the biophysical data obtained for rVes v 5 and the enzymatic activity of rVes v 1 clearly suggest proper folding of both insect cell produced proteins.

**Figure 3:**
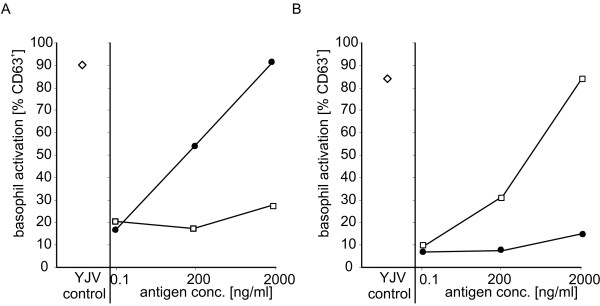
**Basophil activation**. Basophils from two YJV-sensitised patients (A and B) were stimulated with 0.1-2000 ng/ml of rVes v 1 (open squares) and rVes v 5 (filled circles). Activation was determined by CD63 upregulation in FACS. Control stimulation was performed with 50 ng/ml YJV (diamonds).

To verify these data in a cell based approach, human basophils isolated from whole blood of venom allergic patients were stimulated with rVes v 1 and rVes v 5, whereby the inactive rVes v 1 S137G/N165A mutant was employed to avoid unspecific basophil activation through phospholipase activity. Stimulation with YJV at a concentration of 50 ng/ml served as control. Clear cellular activation as quantified by CD63 upon stimulation with the recombinant allergens could be observed over a concentration range from 0.1 ng/ml to 2 μg/ml (Fig. 3; additional file 3).

Together, these data support the applicability of the recombinant proteins for cellular approaches in hymenoptera venom diagnosis and moreover corroborate the idea that insect cells are an ideal host for expression of hymenoptera venom proteins.

### Immunoreactivity of rVes v 1 and rVes v 5

To reevaluate the immunoreactivity and diagnostic relevance of Ves v 1 and Ves v 5 on a molecular, component resolved level, individual patient sera of 34 patients with a positive sIgE test to either HBV and YJV, or YJV only, were assayed by ELISA for specific IgE antibodies. To further provide a broadened reactivity profile and allow for assignment of sensitisation, nApi m 1, considered a surrogate marker for sensitisation to *A. mellifera *venom, and the CCD marker MUXF-BSA that provides the core fucosylated glycotope isolated from pineapple stem bromelain [13] were included.

**Figure 4:**
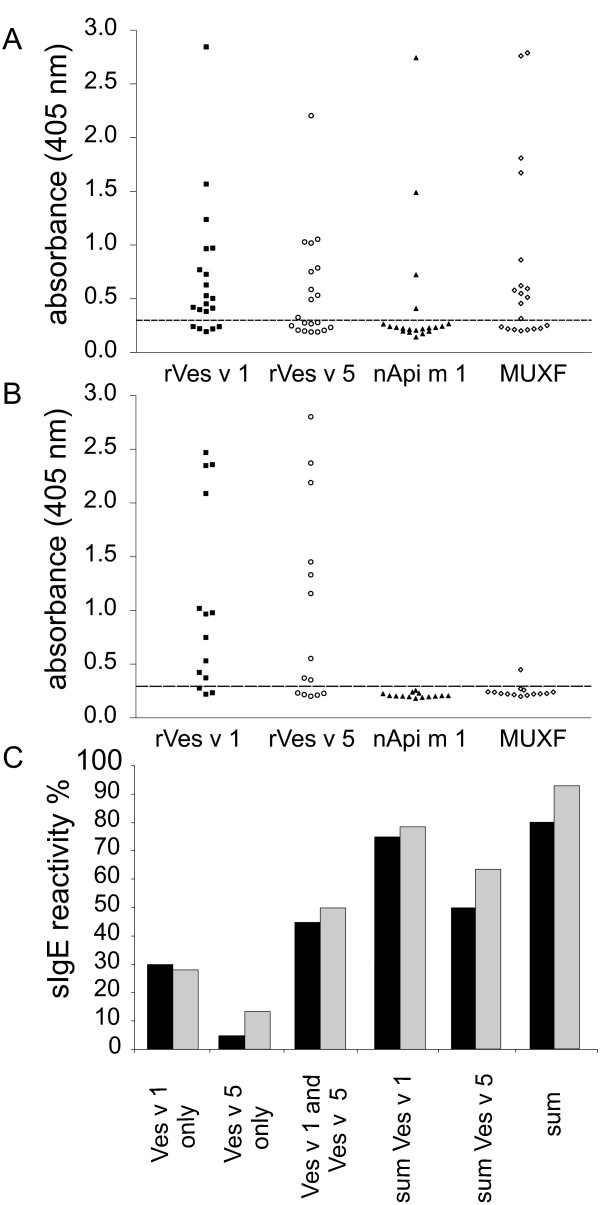
**IgE reactivity of patient sera**. IgE reactivity of individual patient sera from double-positive (A) or YJV-positive patients (B) to rVes v 1 and rVes v 5 produced in Sf9 insect cells, nApi m 1, and MUXF-BSA. The cut-off of the ELISA is indicated by a dashed line (mean background plus two fold the SD). The percentage of sIgE reactivity to either each allergen (Ves v 1 only/Ves v 5 only) or both allergens (Ves v 1 and Ves v 5) is represented in C for double-positive (black bars) or YJV-positive (grey bars) patients. The overall sIgE reactivity to Ves v 1 or Ves v 5 (sum Ves v 1 or sum Ves v 5) was obtained by addition of exclusive sIgE reactivity to one allergen to sIgE reactivity if not recognized exclusively. Overall diagnostic sensitivity (sum) was obtained by addition of all sIgE reactivities.

Of the 20 double-positive sera (Fig. 4A; additional file 4) 15 showed reactivity to rVes v 1, 10 of which additionally had specific IgE to rVes v 5. Interestingly, only 1 out of these 20 sera had sIgE to rVes v 5 exclusively, while 2 sera exhibited additional reactivity to Api m 1. In summary, in this group an overall diagnostic sensitivity of 80% could be achieved by use of two YJV allergens, compared to 50% when using rVes v 5 solely (Fig. 4C). Of the remaining 4 patients 2 had sIgE for nApi m 1 and 1 was reactive to the CCD marker MUXF-BSA only. Thus, for 16/20 patients (80%), a particular culprit venom could convincingly be assigned (Fig. 4C) whereas 2 patients showed a true double-sensitisation. Only 1 patient showed reactivity neither to Ves v 1 nor to Ves v 5. Notably this patient also showed no reactivity to other vespid proteins such as the hyluronidases Ves v 2a and b and the dipeptidylpeptidase Ves v 3 (data not shown).

In an additional HBV mono-sensitised control group 4 out of 5 patients had sIgE to Api m 1. As anticipated none showed reactivity to the yellow jacket allergens and the CCD marker MUXF-BSA (data not shown).

In the YJV mono-sensitised group (Fig. 4B; additional file 4) 11 of 14 sera (79%) were reactive to rVes v 1, 7 of which exhibited additional sIgE reactivity to rVes v 5. Two further patients showed sIgE reactivity exclusively to rVes v 5. Thus, in summary 13/14 (93%) had detectable sIgE either to rVes v 1, rVes v 5 or both (Fig. 4C) while 1 patient with low total YJV sIgE showed no reactivity. Assessment of this serum for specific IgG provided no evidence for the presence of blocking IgG antibodies (data not shown). In accordance with the sensitisation of this group, no reactivities to nApi m 1 were observed. However, 1 patient exhibited low sIgE reactivity to the CCD marker.

These data demonstrate that recombinant Ves v 1 is an essential component to assess the sensitisation of individuals to YJV and its recombinant availability complemented with Ves v 5 and Api m 1 allows for clear assignment of sensitisation patterns.

## Discussion

Standard diagnostic approaches in hymenoptera venom allergy, but also in plant associated allergies are often hampered by multiple IgE reactivities affecting the interpretation of ambiguous results and the correct choice of the proper venom for immunotherapy, a prerequisite for efficient therapy [1].

Causative for this phenomenon is IgE binding either to peptide epitopes of closely related or homologous proteins or to conserved carbohydrate structures of related or otherwise unrelated glycoproteins. Obviously, the more common incidence in hymenoptera venom allergy is the latter one, the molecular basis of which could be attributed to α-1,3-core-fucose and, exclusively in plants [14], β-1,2-xylose. These residues are absent in mammalian glycosylation and, therefore, constitute a highly immunogenic epitope in men. In contrast to protein-directed cross-reactivity, the carbohydrate-directed reactivity in food and hymenoptera allergy is mainly believed to be clinically irrelevant, but diagnostically cumbersome [15,16].

Since identification of the culprit venom is strongly affected by such double positivities the choice of the proper venom for immunotherapy is often difficult. A sophisticated method to identify and circumvent such reactivities are inhibition tests based on mutual inhibition of IgE by venom of the particular species [17]. However, these tests are not widely used in standard diagnosis and the obtained data are difficult to interpret. Hence, the more advanced and promising option relies on the use of unique recombinant major allergens which are representative for the respective venom and fulfil all criteria regarding high prevalence and low cross-reactivity of both types.

For HBV, phospholipase A2 (Api m 1) is considered an ideal surrogate marker as it is structurally unrelated to the phospholipase A2 in vespid venom and shows a high prevalence of sIgE recognition [18]. Nevertheless, due to its nature as glycoprotein cross-reactivity on CCD level is possible, even if this seems to be reduced in a natural conformation as indicated by patient sera that showed IgE reactivity to MUXF and glycoproteins of insect origin (data not shown) but not to Api m 1. No other proteins in HBV fit to the diagnostic needs due to presence of multiple glycosylation sites and confirmed CCD reactivities. However, recombinant approaches might offer opportunities for establishment of improved allergen molecules devoid of glycosylation.

In contrast, YJV contains two non-glycosylated major allergens without cross-reactive homologues in other species, Ves v 1 and Ves v 5, both showing high IgE prevalence, as shown for proteins purified from venom [19]. Allergens like Ves v 2 a and b and Ves v 3 do not meet those criteria as they are glycoproteins and in case of the Ves v 2 isoforms show only minimal IgE prevalence apart from carbohydrate based IgE binding. In contrast, the HBV hyaluronidase Api m 2 appears to be a true, but minor allergen recognised by approx. 30% of sensitised patients only [7,8]. Furthermore, the dipeptidylpeptidases from HBV and YJV have been shown to be cross-reactive due to their high sequence homology [8].

The phospholipase A2 from HBV can be purified from venom that is easily obtained by electrostimulation, nevertheless, recombinant production could be shown in bacteria. Although refolded from insoluble aggregates enzymatic as well as biological activity of the recombinant phospholipase in terms of effector cell activation proved to be comparable to the native protein [20].

In contrast to Api m 1 and Ves v 5 [9,21] and to the best of our knowledge, no expression of functional phospholipase A1 from YJV has been reported so far [10]. This is even more important since the purification from natural sources is hampered by the significantly higher effort to obtain substantial amounts of YJV.

However, the approach used in this study, production of hymenoptera venom allergens in a nearly autologous system, yielded Ves v 1 for the first time as a soluble and enzymatically active molecule. Interestingly, expression of the active enzyme appears not to be detrimental for the host cells, as shown with the inactive variant of Ves v 1. This finding might be contributed to the lytic nature of the baculovirus mediated overexpression, resulting in apoptosis 48-72 h after infection. Biophysical and biochemical measurements of both recombinant Ves v 1 and Ves v 5 were entirely in accordance with activity and native folding. Under the aegis of insect cell-mediated folding and expression the allergenic characteristics were compatible even with activation of human basophils, an *ex vivo *assay considered to reflect the pathophysiological situation.

By the use of such defined rVes v 1 and rVes v 5 molecules a true sensitisation to YJV for ≥ 80% of the patients independently of their sensitisation could be confirmed, while a true double-sensitisation was verified in only 10% of the double-positive patients as indicated by additional sIgE reactivity to nApi m 1. Thereby, the prevalence of CCD reactive patients was approx. 60% in the double-positive cohort which is in accordance with the literature [1] and can be assumed the only reason for cross-reactivity. In depth follow up studies with increased patient numbers in the future can provide detailed information about the prevalence of IgE reactivity as well as clinical relevance of Ves v 1 and Ves v 5. Additionally, the potential need for further vespid allergens and implementation of the allergens into laboratory systems for quantitative sIgE measurements to enable fail-free detection of all YJV sensitised individuals should be thoroughly evaluated.

## Conclusions

In summary, the use of the defined recombinant major allergens Ves v 1 and Ves v 5 provides a significant improvement for the identification of the culprit venom which is indispensable for the choice of the appropriate immunotherapeutic strategy. Thereby, the need of Ves v 1 for detection of true sensitisation to YJV could be established. Implementation of rVes v 1 to routine diagnosis thus can allow for assessing its true IgE prevalence and clinical relevance beyond estimations from immunoblot studies. Furthermore, component-resolved diagostics using recombinant allergens such as Ves v 1 and Ves v 5 may provide new insights into the role and relevance of particular venom compounds during sensitisation and hyposensitization.

## Competing interests

The authors declare that they have no competing interests.

## Authors' contributions

HS and SB carried out the molecular genetic work, performed the activity tests and the immunoreactivity analyses and drafted the manuscript. LC performed the basophil activation tests. IB participated in the design and drafting of the study and evaluation of the clinical data. RB and TG participated in the design of the study. MO and ES conceived of the study, and participated in its design and coordination and helped to draft the manuscript. All authors read and approved the final manuscript.

## Supplementary Material

Additional file 1 **DLS measurement of rVes v 5**. Dynamic light scattering measurements were carried out using the Spectroscatterer 201 (RiNA GmbH). Protein concentration of rVes v 5 was 0.12 mg/ml in 50 mM sodium phosphate, pH 7.6. rVes v 5 exhibited clear monodispersity with a hydrodynamic radius of 2.6 +/- 0.41 nm. Click here for file

Additional file 2 **Circular dichroism spectroscopy of rVes v 5**. The CD spectrum for rVes v 5 with a minimum at 208 nm and a shoulder at 225 nm was superimposable to data reported for native Ves v 5. Click here for file

Additional file 3 **Serological data of patients assessed in basophil activation**. sIgE levels for HBV (i1) and YJV (i3) were determined with the Immulite 2000 (Siemens Healthcare Diagnostics). Click here for file

Additional file 4 **Serological data of patients assessed in IgE reactivity analysis**. The sIgE levels for HBV (i1) and YJV (i3) were determined with the Immulite 2000 (Siemens Healthcare Diagnostics) or ImmunoCap 250 (Phadia). In singular cases sIgE values were not determined, but patients were positive in skin prick testing. For some patients sIgE values are expressed by classes according to the manufacturer. The sIgE values to rVes v 1 and rVes v5 were considered as positive (+) at a OD 405 nm > 0.27. Cut off for high sIgE levels (++) was at OD 405 nm > 1. Click here for file
