# Supplementary figures and images for: Recombinant phospholipase A1 (Ves v 1) from yellow jacket venom for improved diagnosis of hymenoptera venom hypersensitivity
Source: Clin Mol Allergy. 2010 Apr 1;8:7. doi: 10.1186/1476-7961-8-7 (PMC2867971; doi:10.1186/1476-7961-8-7)

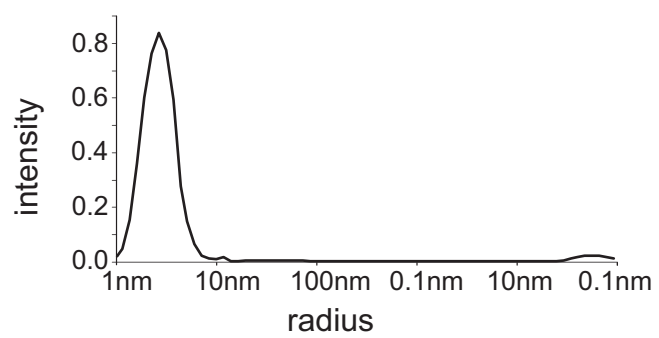

Supplement: Additional file 1 — DLS measurement of rVes v 5. Dynamic light scattering measurements were carried out using the Spectroscatterer 201 (RiNA GmbH). Protein concentration of rVes v 5 was 0.12 mg/ml in 50 mM sodium phosphate, pH 7.6. rVes v 5 exhibited clear monodispersity with a hydrodynamic radius of 2.6 +/- 0.41 nm. [file 1476-7961-8-7-S1.PDF]

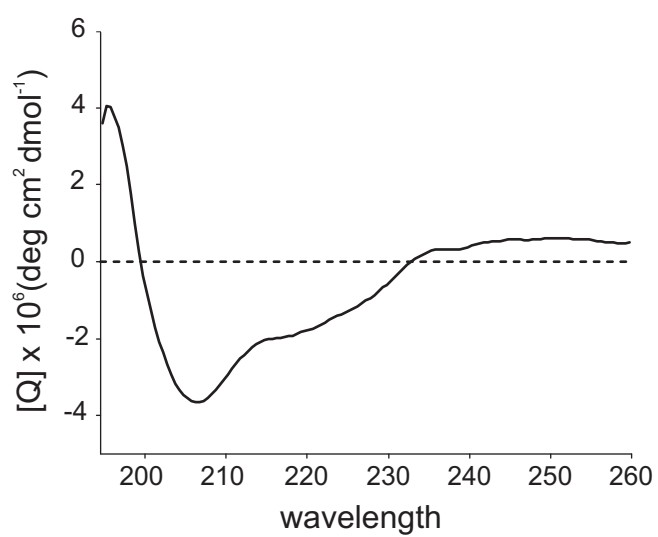

Supplement: Additional file 2 — Circular dichroism spectroscopy of rVes v 5. The CD spectrum for rVes v 5 with a minimum at 208 nm and a shoulder at 225 nm was superimposable to data reported for native Ves v 5. [file 1476-7961-8-7-S2.PDF]
