# Supplementary material for: Recombinant phospholipase A1 (Ves v 1) from yellow jacket venom for improved diagnosis of hymenoptera venom hypersensitivity
Source: Clin Mol Allergy. 2010 Apr 1;8:7. doi: 10.1186/1476-7961-8-7 (PMC2867971; doi:10.1186/1476-7961-8-7)
Supplement: Additional file 3 — Serological data of patients assessed in basophil activation. sIgE levels for HBV (i1) and YJV (i3) were determined with the Immulite 2000 (Siemens Healthcare Diagnostics). [file 1476-7961-8-7-S3.DOC]

| patient |  | |
| --- | --- | --- |
|  | HBV (i1)  (kU/L) | YJV (i3)  (kU/L) |
| A | 0.82 | 5.84 |
| B | n.d. | 6.92 |
