# Supplementary material for: Recombinant phospholipase A1 (Ves v 1) from yellow jacket venom for improved diagnosis of hymenoptera venom hypersensitivity
Source: Clin Mol Allergy. 2010 Apr 1;8:7. doi: 10.1186/1476-7961-8-7 (PMC2867971; doi:10.1186/1476-7961-8-7)
Supplement: Additional file 4 — Serological data of patients assessed in IgE reactivity analysis. The sIgE levels for HBV (i1) and YJV (i3) were determined with the Immulite 2000 (Siemens Healthcare Diagnostics) or ImmunoCap 250 (Phadia). In singular cases sIgE values were not determined, but patients were positive in skin prick testing. For some patients sIgE values are expressed by classes according to the manufacturer. The sIgE values to rVes v 1 and rVes v5 were considered as positive (+) at a OD 405 nm > 0.27. Cut off for high sIgE levels (++) was at OD 405 nm > 1. [file 1476-7961-8-7-S4.DOC]

| double-positive | | | | | YJV positive | | | | |
| --- | --- | --- | --- | --- | --- | --- | --- | --- | --- |
| No. | HBV (i1)  (kU/L) | YJV(i3)  (kU/L) | Ves v 1 sIgE | Ves v 5 sIgE | No. | HBV (i1)  (kU/L) | YJV (i3)  (kU/L) | Ves v 1 sIgE | Ves v 5 sIgE |
| 1 | >100 | 20.5 | + | - | 1 | <0.1 | class 4 | ++ | ++ |
| 2 | 6 | 56 | + | + | 2 | <0.1 | >100 | ++ | ++ |
| 3 | >100 | 6.72 | - | ++ | 3 | <0.1 | class 2 | + | + |
| 4 | 20.5 | 1.41 | + | + | 4 | <0.1 | 43.7 | ++ | ++ |
| 5 | 5.2 | 17.9 | + | + | 5 | <0.1 | 5.93 | - | + |
| 6 | 20.6 | 0.56 | - | - | 6 | <0.1 | 8.69 | + | ++ |
| 7 | 0.81 | 18.4 | ++ | + | 7 | <0.1 | 19.4 | ++ | - |
| 8 | 4.04 | 33.4 | + | ++ | 8 | <0.1 | 27 | - | ++ |
| 9 | 0.22 | 16.9 | + | - | 9 | <0.1 | 23.5 | ++ | ++ |
| 10 | 12.6 | 0.56 | - | - | 10 | <0.1 | 75.5 | + | - |
| 11 | 9.93 | 96.2 | ++ | ++ | 11 | <0.1 | 9.61 | + | - |
| 12 | 17.7 | 7.65 | - | - | 12 | <0.1 | 0.46 | - | - |
| 13 | 10.9 | 11.9 | + | - | 13 | <0.1 | 3.25 | + | + |
| 14 | >100 | 1.16 | - | - | 14 | <0.1 | 21.3 | ++ | - |
| 15 | n.d | 4.23 | + | + |  |  |  |  |  |
| 16 | n.d | 10.3 | + | - |  |  |  |  |  |
| 17 | n.d | 1.36 | + | - |  |  |  |  |  |
| 18 | 5.54 | 6.76 | + | ++ |  |  |  |  |  |
| 19 | 0.31 | 30.8 | ++ | + |  |  |  |  |  |
| 20 | 3.86 | 14.1 | + | + |  |  |  |  |  |
